# Phase II Study of Pegvorhyaluronidase Alfa (PEGPH20) and Pembrolizumab for Patients with Hyaluronan-High, Pretreated Metastatic Pancreatic Ductal Adenocarcinoma: PCRT16-001

**DOI:** 10.3390/cancers18030507

**Published:** 2026-02-03

**Authors:** Elena Gabriela Chiorean, Sheela R. Damle, David B. Zhen, Martin Whittle, Ben George, Howard Hochster, Andrew L. Coveler, Andrew Hendifar, Tomislav Dragovich, Rachael A. Safyan, Gentry T. King, William P. Harris, Barbara Dion, Amy Stoll D’Astice, Arthur Lee, Shelley Thorsen, Sita Kugel, Adam Rosenthal, Sunil Hingorani

**Affiliations:** 1 University of Washington School of Medicine, Seattle, WA 98195, USA; sdamle@uw.edu (S.R.D.); dbzhen@uw.edu (D.B.Z.); acoveler@uw.edu (A.L.C.); rsafyan@uw.edu (R.A.S.); gentryk@uw.edu (G.T.K.); wph3@uw.edu (W.P.H.); 2 Fred Hutchinson Cancer Center, Seattle, WA 98109, USAsthorsen@fredhutch.org (S.T.); skugel@fredhutch.org (S.K.); shingorani@unmc.edu (S.H.); 3 Medical College of Wisconsin, Milwaukee, WI 53226, USA; george.ben@mayo.edu (B.G.); badion@mcw.edu (B.D.); 4 Mayo Clinic, Rochester, MN 55905, USA; 5 Rutgers Cancer Institute, New Brunswick, NJ 08903, USA; howard.hochster@rutgers.edu; 6 Cedar-Sinai Medical Center, Los Angeles, CA 90048, USA; andrew.hendifar@cshs.org; 7 Baptist MD Anderson Cancer Center, Jacksonville, FL 32207, USA; tom.dragovich@bmcjax.com; 8 Cancer Research and Biostatistics, Seattle, WA 98109, USA; amystoll2@cox.net (A.S.D.); leearth@gmail.com (A.L.); adamr@crab.org (A.R.); 9 University of Nebraska Medical Center, Omaha, NE 68198, USA

**Keywords:** hyaluronidase, pancreatic adenocarcinoma, PEGPH20, pembrolizumab, stroma, tumor immune microenvironment

## Abstract

**Background**: Stromal hyaluronic acid (HA) poses a physical barrier and protects tumor cells from immune surveillance. Stroma targeting with pegylated human recombinant PH20 hyaluronidase (PEGPH20) demonstrated improved infiltration of cytotoxic T-lymphocytes and delivery of chemotherapy and PD1/PD-L1 antibodies in tumor models. This multicenter phase II study of PEGPH20 plus pembrolizumab evaluated the efficacy, safety and immune and stromal biomarkers in patients with HA-high refractory metastatic pancreatic ductal adenocarcinoma (mPDA). **Patients and Methods**: Patients were treated with PEGPH20 3 µg/kg IV weekly and pembrolizumab 200 mg IV in 3-week cycles. Tumor and blood samples were collected at baseline and on-study for biomarker analyses. **Results:** Between May and November 2019, 38 patients were screened and 8 treated, with median age 68 years (range 60–73) and median two (range 1–4) prior therapies. The study was closed to accrual early by pharmaceutical sponsor. Treatment was well tolerated, with expected grade 1/2 musculoskeletal toxicities. Best response was stable disease in 2 of 7 evaluable patients (29%). Median overall and progression-free survival were 7.2 months (95% CI 1.2–11.8) and 1.5 months (95% CI 0.9–4.4), respectively. Prolonged survival (range 10.2–27.6 months) occurred in patients treated with subsequent chemotherapy. Higher baseline tumor T cell receptor (TCR) clonality correlated with longer survival. **Conclusions**: Pembrolizumab with PEGPH20 was safe but did not have significant efficacy in refractory HA-high metastatic PDA.

## Simple Summary

Pancreatic ductal adenocarcinoma (PDA) is a stroma-rich tumor which poses a barrier to anti-tumor immune surveillance. Stroma targeting with PEGPH20 improves infiltration of cytotoxic T-lymphocytes and delivery of PD1/PD-L1 antibodies in tumor models. This multicenter phase II study evaluated the efficacy, safety, and biomarkers of immune response and stroma modulation in patients with HA-high refractory PDA treated with PEGPH20 and pembrolizumab. Treatment was safe and conferred a median overall survival of 7.2 months in PDA patients pretreated with a median of two prior therapies. Nevertheless, with best response of stable disease in two of seven patients (29%) and PFS of only 1.5 months, this regimen was not deemed sufficiently effective in refractory HA-high PDA. Biomarker analysis noted a correlation between higher baseline tumor T cell receptor (TCR) clonality and longer overall survival.

## 1. Introduction

Pancreatic ductal adenocarcinoma (PDA) is notoriously treatment-resistant and has an immunosuppressive tumor microenvironment (TME) which contributes to it being among the most aggressive malignancies. Patients with metastatic PDA progressing after first-line chemotherapy have a median overall survival (OS) of 5–6 months and progression-free survival (PFS) of 2.5–3 months with second-line regimens [1,2,3]. Furthermore, no standard treatment exists after two lines of therapy [4]. PDA may overexpress programmed death-ligand 1 (PD-L1), and the degree of PD-L1 expression correlates with tumor stage and increased regulatory T cell (T_reg_) infiltration [5,6]. Despite PD-1 blockade inducing tumor regression in pancreatic tumor models, PD-1/PD-L1 immune checkpoint inhibitors (ICI) have no significant clinical activity in PDA [7]. Nevertheless, some reports suggest that combinatorial approaches with chemotherapy, radiotherapy, CD40 agonists or CXCR4/CXCL12 inhibitors have antitumor efficacy associated with effector T cell activation and infiltration [8,9,10,11]. Microsatellite instability-high (MSI-high) and high tumor mutational burden (TMB) above 10 mutations/megabase (m/Mb) have benefitted from ICI in solid tumors, but efficacy appears to be lower in patients with PDA [12]. Less than 1% of PDA is MSI-high, and only 1% has TMB higher than 10 m/Mb, which often coincides with MSI-high status. While TMB is typically less than 3 m/Mb in PDA [13], TMB higher than 20 m/Mb is thought to be needed to confer sensitivity to ICI for patients with PDA [14].

Pancreatic epithelial and stromal cells secrete high levels of hyaluronan (hyaluronic acid, HA) which poses a physical barrier within the extracellular matrix to therapeutic agents [15,16,17,18]. In addition, HA influences immune surveillance by inducing monocyte differentiation into fibrocytes and converts effector memory CD4^+^ T cells to IL-10-producing T_regs_ [19,20]. PH20 hyaluronidase (pegvorhyaluronidase alfa, PEGPH20), an enzyme capable of depleting HA, can remodel the tumor stroma, thereby allowing infiltration of cytotoxic T lymphocytes and NK cells, and increases intratumoral access for chemotherapeutics and antibodies [15,18,21]. In preclinical models, including PDA, PEGPH20 enhanced Salmonella-based therapy targeting indoleamine 2,3-dioxygenase (shIDO-ST) plus anti-PD-1-mediated tumor regression [21,22]. PEGPH20 showed promising results when combined with gemcitabine (Gem) or with gemcitabine plus nab-paclitaxel (Gem/nabP) in phase I and II clinical trials [23,24], but in the phase III randomized study, HALO-301 in PDA with high HA expression ultimately did not demonstrate improved survival compared to Gem/nabP alone [25]. We considered that combination strategies with stroma-targeting therapies and immune checkpoint blockade could induce synergistic anti-cancer activity and immune responses in pancreatic cancer. This phase II trial aimed to determine the efficacy and safety of pembrolizumab plus PEGPH20 in pretreated patients with metastatic PDA with HA-high expression, as well as to test stroma- and immune-related biomarkers and correlate these with clinical benefits.

## 2. Materials and Methods

### 2.1. Patient Eligibility

Eligible patients were ≥18 years, with histologically proven metastatic PDA, ECOG performance status of 0 or 1, prior treatment with up to two lines of therapy for metastatic disease, life expectancy ≥ 12 weeks, and adequate organ function. Patients must have been amenable to undergo baseline and on-study tumor biopsies for research purposes, if safe and feasible. Patients were required to have hyaluronan-high tumors, defined as ≥50% HA staining of any intensity in the tumor extracellular matrix (ECM) analyzed centrally with a hyaluronan affinity histochemistry assay (Ventana HA RxDx Assay; Roche, Tucson, AZ, USA). Exclusion criteria included recent history (within 4 weeks) of arterial or venous thromboembolic events, prior therapy with anti-PD-1/PD-L1 agents or with PEGPH20, brain metastases, and contraindications to heparin or to study agents. All patients provided written informed consent approved by the Fred Hutchinson Cancer Center Institutional Review Board (IRB) as well as the local IRBs of the participating institutions (Medical College of Wisconsin, Rutgers Cancer Institute). The Cedars–Sinai Cancer Center and Banner MD Anderson Cancer Center were in the process of study activation at the time the study closed to accrual. The study was conducted in accordance with the International Conference on Harmonization Good Clinical Practice guidelines. The trial is registered at ClinicalTrials.gov (ClinicalTrials.gov identifier: NCT03634332).

### 2.2. Study Design and Treatments

This was a phase II open-label, single-arm, non-randomized multicenter study conducted through the Pancreatic Cancer Research Team (PCRT)—Cancer Research and Biostatistics (CRAB) consortium. Patients were initially pre-screened for tumor HA expression from archival tumor specimens. Archival tumor tissue with enough tumor presence to make a minimum of 5–10 unstained, consecutive formalin-fixed paraffin embedded (FFPE) slides or one archival block was required for HA testing using the Ventana HA RxDx assay [24]. If tumors tested were HA-high and patients were deemed eligible for study treatment, they were enrolled in the study. Patients with tumors which were not HA-high screen-failed.

Pembrolizumab 200 mg was administered intravenously (IV) on day (D) 1 and PEGPH20 3 µg/kg was administered IV on D1, D8, and D15 in 21-day cycles. On concurrent dosing days, pembrolizumab was dosed 2–4 h after PEGPH20. To prevent and treat musculoskeletal events (MSEs) from PEGPH20, non-steroidal anti-inflammatory agents (NSAIDs) such as piroxicam 20 mg or ibuprofen 400 mg were administered 1–2 h prior to and 8–12 h after PEGPH20 dosing. Prophylactic use of proton pump inhibitors, such as omeprazole 20 mg daily, was recommended for all patients receiving piroxicam. Steroids (prednisone 5–10 mg or dexamethasone 2–4 mg) could be used for intolerable MSEs from PEGPH20, or for patients unable to tolerate NSAIDs, within 2 h prior to and 8–12 h after PEGPH20. NSAIDs and steroids could be tapered down/off after cycle 2 if no significant MSEs occurred. To minimize the risk of thromboembolic events associated with PEGPH20, enoxaparin 1 mg/kg/day subcutaneously or direct oral anticoagulants were self-administered by all patients. Patients who discontinued anticoagulants were required to discontinue PEGPH20. Study treatment continued until disease progression, unacceptable toxicity, withdrawal of consent or study termination by pharmaceutical sponsors.

Fresh tumor biopsies (four cores from metastatic lesions or from primary tumor) for research purposes were to be obtained pre-treatment at baseline (enrollment) and on cycle 3 D1 (±3 days), if safe and feasible, and optionally at time of treatment discontinuation. If tumor samples were insufficient, biomarker analyses were prioritized as follows: (1) T cell receptor (TCR) sequencing, (2) flow cytometry for immune phenotyping. Blood samples for research purposes were planned pre-treatment (plasma HA levels, TCR sequencing, flow cytometry), prior to dosing in each cycle (plasma HA levels), cycle 3 D1 (±3 days) (TCR sequencing, flow cytometry), and at treatment discontinuation (plasma HA levels, TCR sequencing, flow cytometry). Archival tumor tissue was used to determine HA and PD-L1 status. Results of tumor molecular alterations from next-generation sequencing (NGS) assays were collected, when available.

### 2.3. Biomarker Analyses

#### 2.3.1. Plasma HA

Blood samples for plasma HA analyses collected at baseline, prior to dosing on day 1 of each cycle, and at the time of treatment discontinuation were analyzed at a bioanalytical laboratory (MicroConstants, Inc., San Diego, CA, USA), using a validated assay [26]. Plasma concentrations of HA were determined using a lower limit of quantification of 42.3 ng/mL.

#### 2.3.2. TCR Sequencing

TCR sequencing and clonality quantification were evaluated from peripheral blood mononuclear cells (PBMCs) and from tumor samples using the ImmunoSEQ^®^ platform (Adaptive Biotechnologies^TM^, Seattle, WA, USA) [27,28]. Blood samples were processed as previously described. Briefly, blood was collected in 60 mL heparin-coated syringes (Becton, Dickinson and Company, Franklin Lakes, NJ, USA). PBMCs were isolated by Ficoll (GE Healthcare, Chicago, IL, USA) separation, washed in media, frozen in 10% DMSO (Sigma-Aldrich, Burlington, MA, USA), and stored in liquid nitrogen until testing. For each patient, PBMC samples were thawed and frozen as a cell pellet, ensuring that the same number of cells was sent for each time point. PBMC samples from 87 healthy donors were obtained from Adaptive Biotechnologies TCRB-V4b control database (https://doi.org/10.21417/ADPT2020V4CD (accessed on 23 July 2019)) and used as controls. Tumor samples were preserved using RNAlater (Qiagen, Venlo, The Netherlands) and stored at −80 °C. Tumor DNA was isolated by mincing followed by extraction utilizing a DNeasy kit (Qiagen). T cell clonality was evaluated from single cell suspensions of flash frozen tumor samples using the ImmunoSEQ^®^ platform (Adaptive Biotechnologies). T cell numbers were determined by flow cytometry. Any sample with fewer than 100 estimated T cells present was discarded for this analysis. We obtained on average between 19,895 and 127,131 productive TCR reads per sample. Productive Simpson clonality, calculated for a sample as the square root of Simpson’s diversity index for all productive re-arrangements [29], maximum productive frequency, and clone frequency were assessed in tumor and PMBC samples at indicated time points.

#### 2.3.3. Immune Phenotyping by Flow Cytometry

To isolate PBMCs, whole blood was collected in heparin green top tubes. Fresh tumor samples were stored in MACS tissue storage solution at 4 °C (Miltenyi Biotec, Bergisch Gladbach, Germany), within 1–16 h post-collection. Tumor tissue was mechanically disrupted into 1–2 mm pieces and digested using the Tumor Dissociation Kit (Miltenyi Biotec) and gentleMACS^TM^ Dissociator (Miltenyi Biotec) following the manufacturer’s recommendations. Tissue and digestion media (RPMI with 10% FBS, 10 mM Hepes, 5 mM CaCl_2_ 1× protease inhibitor cocktail, 1× trypsin inhibitor and 100 U/mL DNase) were heated to 37 °C for 20–40 min until homogenization was achieved. Tumor tissues were filtered 3 times using a 70 μM cell strainer to remove undigested tissue and dead cell debris and resuspended in 1 mL of PBS. The resulting single cell suspension was immediately analyzed by flow cytometry.

T cells isolated from PBMCs and tumors were labeled with antibodies for CD3 (clone UCHT1, BD Biosciences, Seattle, WA, USA), CD4 (clone SK3, BioLegend, San Diego, CA, USA), and CD8 (clone RPA-T3, BD). For each subset, differentiation (naïve, central memory, effector memory) and activation status were assessed with markers for CD279/PD-1 (clone EH12.2H7, BioLegend), CD223/LAG3 (clone 11C3C65, BioLegend), TIM3 (clone F38-2E2, BioLegend), FOXP3 (clone 206D, BioLegend), CD45RA (clone HI100, BioLegend), CD45RO (clone UCHL1, BioLegend), CD187/CCR7 (clone G043H7, BioLegend), CD25 (clone M-A251, BioLegend), Granzyme B (clone QA16A02, BioLegend), and HLA-DR (clone L243, BioLegend). A fixable viability dye was used to exclude dead cells (Fixable Viability Dye eFluor 780, eBioscience, San Diego, CA, USA). Banked PBMC samples were available from untreated pancreatic cancer patients (n = 6) and a healthy donor (n = 1) and were used as controls.

#### 2.3.4. PD-L1

Archival tumor samples were tested for PD-L1 expression by immunohistochemistry (IHC), using the Merck 22C3 antibody according to the manufacturer’s instructions. Five unstained slides cut from an FFPE block were submitted immediately after sectioning to QualTek Molecular Laboratories (Santa Barbara, CA, USA). PD-L1 expression was scored using the modified proportion score (MPS) counting PD-L1 expressing tumor cells and mononuclear inflammatory cells. To be evaluable for PD-L1 testing a sample had to have at least 50 viable neoplastic cells or at least 5 viable PD-L1-positive neoplastic cells.

All biomarker analyses, unless otherwise specified, were performed in Dr. Sunil Hingorani’s Laboratory at the Fred Hutchinson Cancer Center, Seattle, WA, USA.

### 2.4. Statistical Methods

Patients were enrolled by the treating physicians in this open-label, single-arm, non-randomized study at participating cancer centers. Based on historical data with second-line treatments for metastatic PDA conferring median PFS of 3 months [1,2,3], we considered the null hypothesis for PFS to be 3 months in this study, which allowed patients to be treated in the second line and beyond. For the primary endpoint of PFS, with a total sample size of 31 patients and a one-sided alpha-level of 0.05, assuming 12 months for accrual and a minimum of 6 months of follow-up, the study had approximately 80% power to detect a difference between the null hypothesis of median PFS of 3 months, versus the alternative hypothesis of median PFS of 6 months. Using a one-sided alpha of 0.05, we would reject the null hypothesis of 3 months if we observed a median PFS of at least 4.84 months (minimum HR of 1.613 to reject the null hypothesis based on the assumption that progressions are exponentially distributed). For OS and PFS, censoring occurred at the date of last contact.

The primary endpoint of PFS was defined as time from registration to disease progression, symptomatic deterioration, or death by any cause. Secondary endpoints included safety and tolerability, overall response rate (ORR) defined as confirmed and unconfirmed complete (CR) and partial response (PR), disease control rate (DCR) defined as confirmed and unconfirmed CR and PR and confirmed and unconfirmed stable disease (SD), duration of response (DoR) defined as time from first documentation of response to PFS failure, and OS defined as time from registration to death from any cause. Responses were tabulated. PFS and OS were estimated using the Kaplan–Meier method.

Safety assessments were conducted weekly throughout the study. Adverse events (AEs) were graded using Common Terminology Criteria for Adverse Events (CTCAE), version 4.0. Tumor response was assessed every 9 weeks (3 cycles), by computed tomography or magnetic resonance imaging, according to modified Response Evaluation Criteria in Solid Tumors (RECIST) version 1.1 [30].

For exploratory biomarker studies, the data was summarized using means and standard deviations, medians and ranges, or percentages as appropriate. Comparisons between baseline and post-treatment continuous factors were performed using Wilcoxon rank sum tests, transforming data where appropriate. The association between changes in correlate values from baseline to post-treatment and best response, PFS or OS was to be evaluated using a Cox proportional hazards model. Student’s *t* test was used to compare TCR clonality indices between patients with short (≤4 months) versus long (>10 months) OS.

## 3. Results

### 3.1. Patients

Thirty-eight patients had archival tumors tested for HA expression between May 2019 and November 2019, of whom 30 were deemed HA-low, and eight patients with HA-high metastatic PDA were eligible and enrolled in the study. The study was closed to accrual early by Halozyme Pharmaceuticals when results from the HALO-301 phase III trial showed no survival advantage with the addition of PEGPH20 to gemcitabine/nab-paclitaxel [25]; therefore, only eight of the planned 31 patients were enrolled. Patients’ baseline characteristics are summarized in Table 1. Median age was 68 years (range 60–73), and patients were treated with a median of 2 (range 1–4) prior lines of therapy. Four patients (50%) were treated with additional chemotherapy after study treatment discontinuation. Median follow-up was 18.6 months.

### 3.2. Safety and Toxicity

Patients completed a median of 2 cycles of treatment (range 1–6). Reasons for study treatment discontinuation were disease progression (n = 4), study termination by pharmaceutical sponsor (n = 3), and patient withdrawal to enroll in hospice (n = 1). No patient discontinued due to adverse events. Of three patients who discontinued treatment due to study termination by sponsor, two had disease progression on subsequent imaging.

Seven patients had treatment-emergent adverse events (TEAE) (Table 2). TEAE occurring in more than one patient were myalgias, edema, muscle cramps, abdominal pain, diarrhea, and vomiting. All TEAE were grade 1 and 2, except for disease-related abdominal pain (n = 2, grade 3), disease-related concurrent biliary obstruction and upper gastrointestinal bleeding (n = 1, grade 3 each), and edema (n = 1, grade 3). Treatment-related adverse events (TRAE) were all grade 1 or 2 except for one grade 3 fatigue (Table 2). TRAE occurring in more than one patient were myalgias (n = 4), peripheral edema and muscle cramps (n = 2 each). Seven serious adverse events occurred in five patients: two patients with abdominal pain due to underlying disease (grade 3); one patient with fever (grade 1), biliary obstruction and upper gastrointestinal hemorrhage due to underlying disease (both grade 3); one patient with pneumothorax from research-related lung biopsy (grade 2); and one patient with treatment-related fatigue (grade 3).

### 3.3. Efficacy

**Figure 1:**
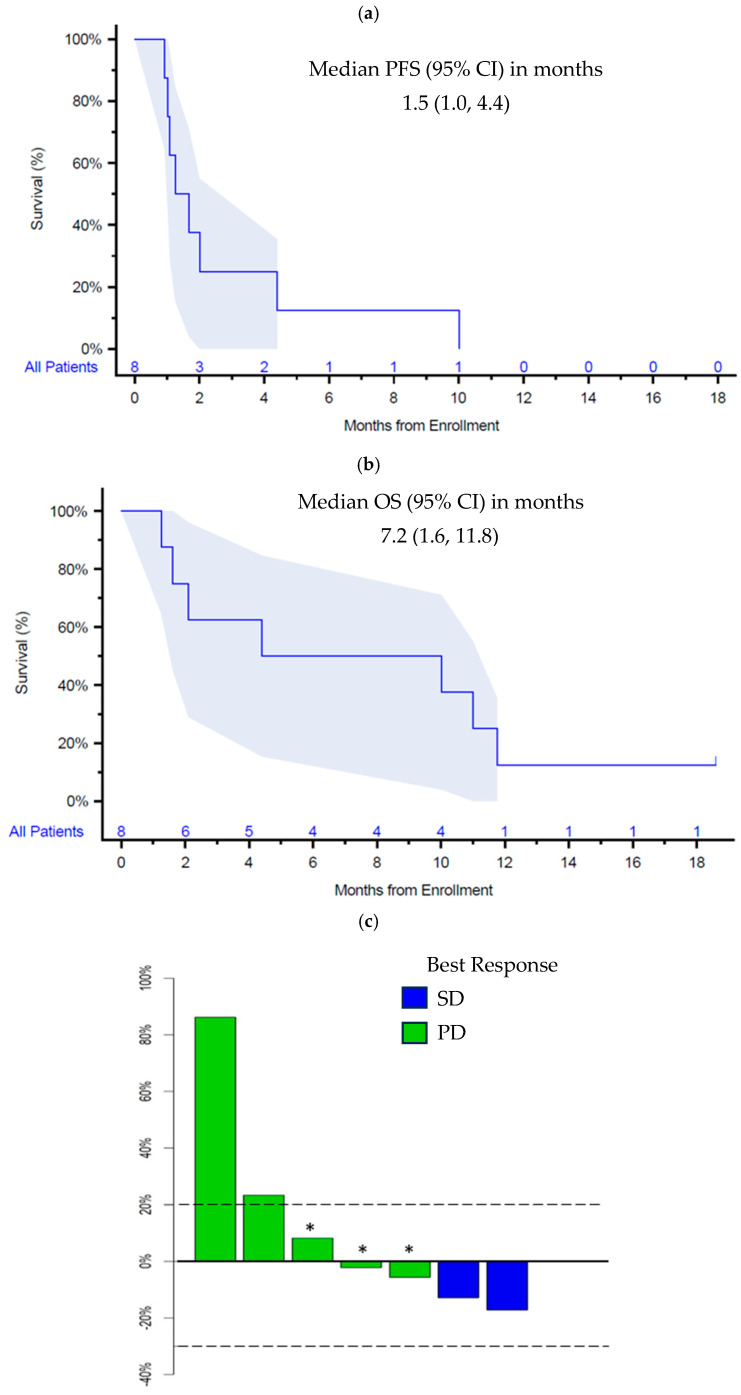
Kaplan–Meier curves of (**a**) progression-free survival and (**b**) overall survival; (c) waterfall plot of best response. Abbreviations: SD, stable disease; PD, progressive disease. Notes: (**a**,**b**) have shaded 95%CI; in Figure 1c patients with PD marked with * had progression in non-target lesions.

Among eight patients enrolled, all were included in survival analyses and seven were evaluable for response. One patient was not evaluable for response due to withdrawal after 2 weeks of treatment to enroll into hospice. Median PFS was 1.5 months (95% CI 1.0–4.4), and median OS was 7.2 months (95% CI 1.6–11.8) (Figure 1a,b). Best response was stable disease (SD) (n = 2, 29%) lasting 9 and 2 months, respectively, and 5 patients (71%) had progressive disease (PD), including three with progression of non-target lesions and two with progression in target lesions (Figure 1c).

Four patients, all with microsatellite stable (MSS) tumors and PD-L1 score < 1, including two patients with SD (one patient each with liver and with lung metastases) and two patients with PD (both had liver and peritoneal metastases), received post-study treatment with chemotherapy and had OS ranging from 10.2 to 27.6 months (Table 3).

### 3.4. Molecular Biomarkers and Correlations with Efficacy

Blood samples for research biomarkers were collected at baseline (n = 8), end of cycle 2 (n = 3), and end of study treatment (n = 2). Tumor samples (liver, n = 4; lung, lymph node, pancreas, peritoneum, n = 1 each) for research biomarkers were collected at baseline (n = 8) and after 6 weeks of treatment (liver, n = 1). Seven patients did not have a second tumor biopsy due to rapid clinical deterioration (n = 4), study termination by pharmaceutical sponsor (n = 2), or safety concerns due to complications from prior research biopsy (pneumothorax, n = 1).

### 3.5. Genomics

Tumor genomic sequencing was performed with FoundationOne^R^ CDx (Foundation Medicine, Cambridge, MA, USA) for four patients. All tumors were MSS and had low TMB (<10 m/Mb), and three were *KRAS*-mutated (Table 3). Two patients did not have the FoundationOne^R^ CDx report available but had a summary of genomic data in the medical record which indicated low TMB < 10 m/Mb, without a discrete value. Among four patients with OS > 10 months, two had available tumor NGS: one patient with *KRAS* wild-type (WT), *RET* fusion-positive, *ATM*-mutated PDA had best response of PD, PFS of 2 months, was treated post-study with nano-liposomal irinotecan and 5-fluorouracil (nal-Iri/5-FU) and had OS of 27.6+ months; one patient with *KRAS* G12R-mutated, *FGFR1*-amplified PDA had best response of PD, PFS of 1.2 months, treated post-study with nal-Iri/5FU and had OS of 12 months. No patient was treated post-study with molecularly targeted therapies.

### 3.6. Plasma HA

**Figure 2:**
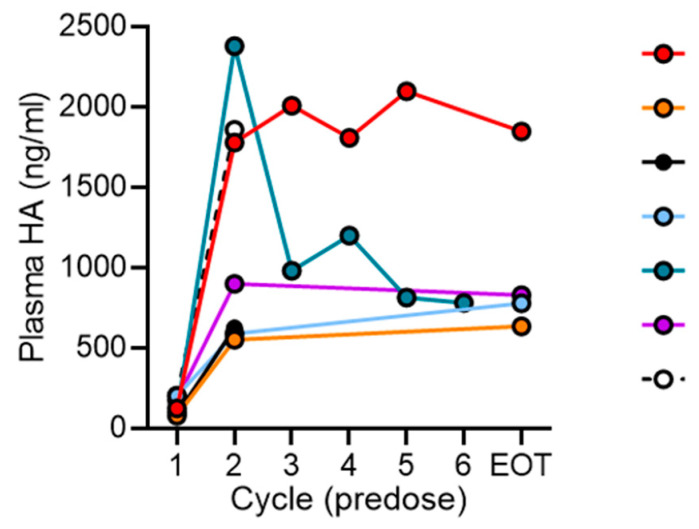
Plasma HA kinetics; each color represents an individual patient. Abbreviations: EOT, end of study treatment; HA, hyaluronic acid.

Plasma HA was analyzed at baseline (n = 8), prior to cycle 2 (n = 7), prior to cycles 3, 4, and 5 (n = 2 each), prior to cycle 6 (n = 1), and at end of treatment (n = 4). Median baseline plasma HA level was 158.5 ng/mL (range 80.8–846). No significant difference was observed between baseline plasma HA for patients with long OS ≥ 10 months [median 114 ng/mL (range 86–204)] compared to short OS < 3 months [median 181 ng/mL (range 80.8–846) (*p*-value = 0.243), or for patients with best response of SD (102 ng/mL, 204 ng/mL) vs. PD (median 158.5 ng/mL (range 80.8–846)] (*p*-value = 0.571). Plasma HA increased 7 to 8-fold during treatment with similar kinetics for patients with long vs. short OS (Figure 2).

### 3.7. TCR Clonality

**Figure 3:**
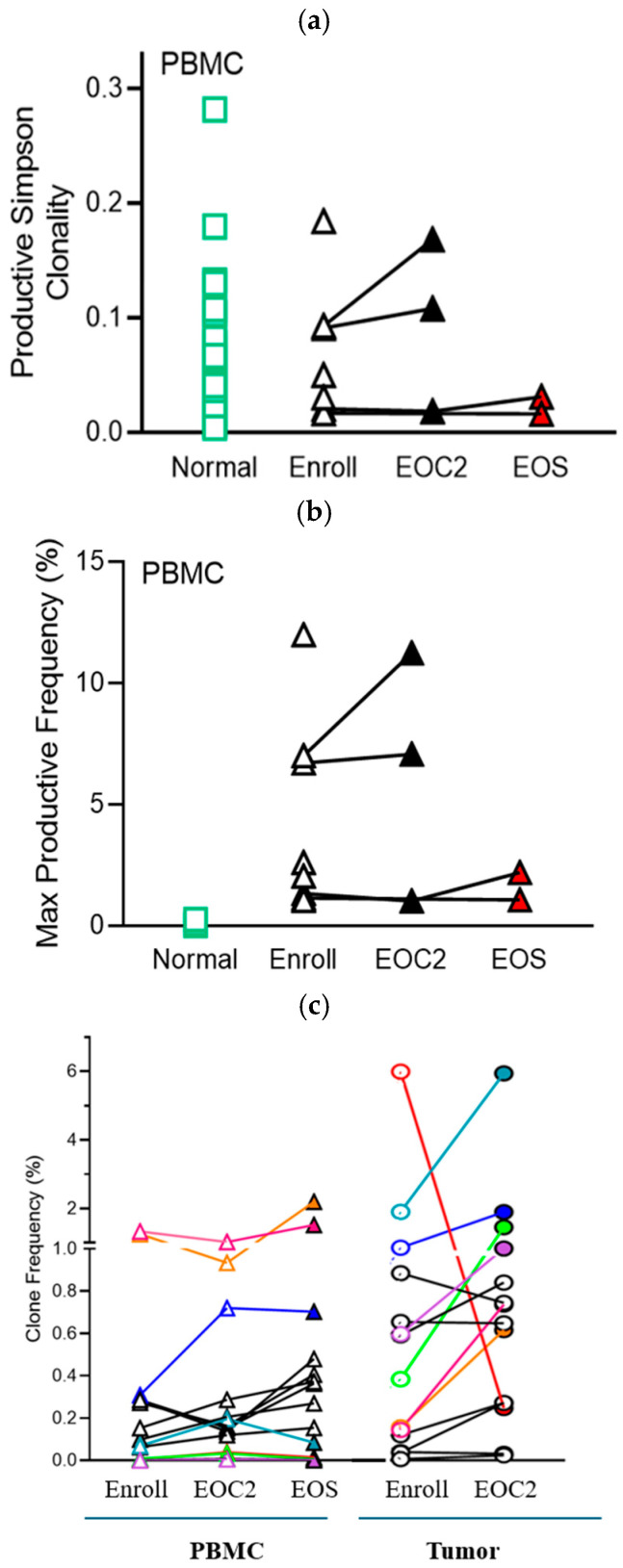

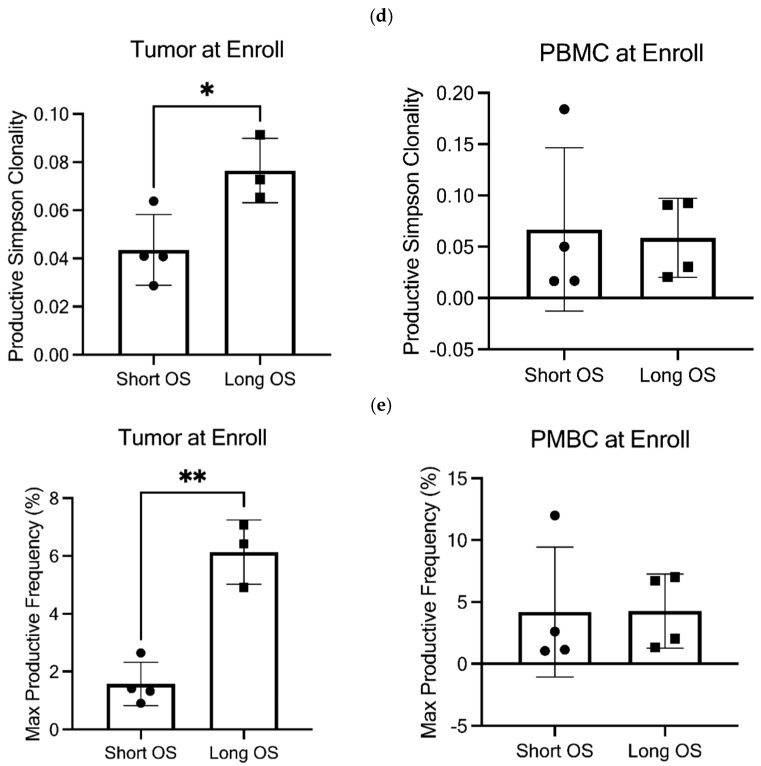
(**a**) TCR clonality in PBMCs during treatment, (**b**) TCR clonal diversity in PBMCs during treatment, and (**c**) TCR clonal diversity during treatment in PBMCs and in tumor of representative patient. (**d**) TCR productive Simpson clonality (mean +/− standard deviation) for tumors and PMBC at time of enrollment stratified by short (≤4 months) versus long (>10 months) OS. (**e**) Maximum (Max) productive frequency (%, mean +/− standard deviation) for tumors and PMBC at time of enrollment stratified by short versus long OS as in (**d**). * *p* < 0.05, ** *p* < 0.01. Note, in (**a**–**c**) triangles represent PBMC samples, and squares represent tumor samples; each color in (**c**) represents an individual TCR clone. Note, one patient in long OS group had no CD8^+^ T cells in tumor at enrollment and was excluded from tumor TCR analysis. Note, in (**d**,**e**) each circle and each square represent an individual sample. Abbreviations: Enroll, baseline/enrollment; EOC2, end of cycle 2; EOS, end of study; PBMCs, peripheral blood mononuclear cells; TCR, T cell receptor; OS, overall survival.

TCR sequencing demonstrated similar fractions of productive, functional TCRs at baseline in patients’ PBMCs and tumors (75–85%) compared to healthy controls (normal, n = 87) (73–90%) (Figure 3a). TCR clonality was generally low at baseline (<0.1), with similar magnitude in peripheral blood and in tumors, and it did not increase significantly during treatment with PEGPH20 and pembrolizumab (Figure 3a). The frequency of productive, expanded TCR clones was significantly higher in PBMC samples from patients with PDA vs. healthy controls (normal) at baseline (Figure 3b), but no significant changes occurred after treatment in the periphery (Figure 3b) or in tumors. For one patient who had paired baseline and on-study biopsies of liver metastasis alongside blood samples, multiple TCR clones were present in PBMCs and in the liver metastasis. While most clones maintained similar frequency in the periphery, some TCR clones significantly expanded intratumorally during treatment (Figure 3c).

Correlative analysis showed that the productive Simpson clonality and the maximum productive frequency of clones in tumors at baseline (after enrollment) were higher in patients with long vs. short OS (Figure 3d,e). This was not observed in peripheral blood samples (Figure 3d,e). No correlations were observed between baseline peripheral or tumor TCR clonality and best response of SD or PD.

### 3.8. Immune Phenotyping

Slightly higher numbers of CD3^+^CD8^+^CD4^−^ cytotoxic T cells and CD45RO^+^/CD45RA^−^ memory CD8^+^ and CD4^+^ T cells were noted in tumors vs. matched PBMCs at baseline (Supplementary Figure S1a–c). Higher numbers of CD3^+^CD8^−^CD4^+^ helper T cells and CD45RO^−^/CD45RA^+^ naïve T cells were present in the periphery vs. tumors at baseline. Only one patient each underwent serial blood or tumor flow cytometry testing during study treatment. For these patients, cytotoxic T cell numbers further increased intratumorally, and memory CD8^+^ and CD4^+^ T cells increased in the periphery but not in tumors during treatment (Supplementary Figure S1a–c).

**Figure 4:**
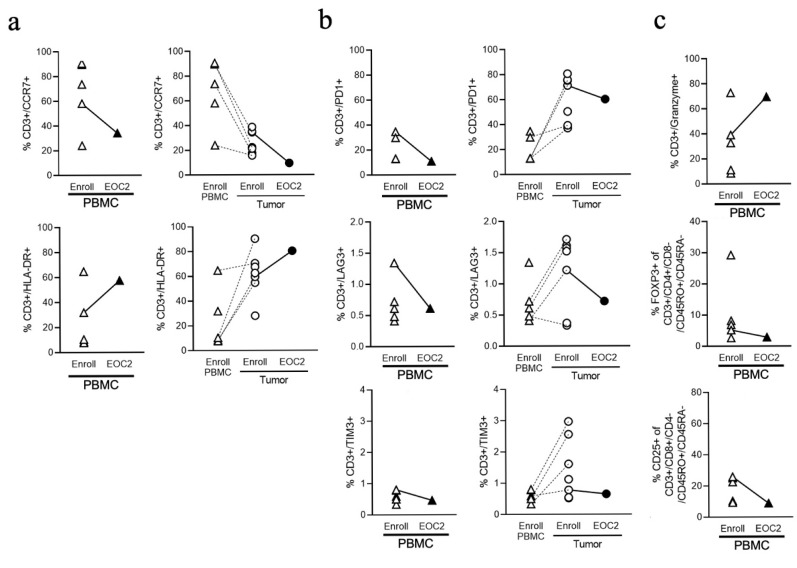
Immune phenotyping for (**a**) CCR7^+^ and HLA-DR^+^ CD3^+^ T cells as a percentage of CD3^+^ T cells from PBMCs and tumors, (**b**) PD-1^+^, LAG-3^+^, and TIM-3^+^ CD3^+^ T cells as a percentage of CD3^+^ T cells from PBMCs and tumors, and (**c**) Granzyme B^+^, FoxP3^+^, and CD25^+^ CD3^+^ T cells in PBMCs. Note, in (**a**–**c**) triangles represent PBMC samples, and squares represent tumor samples Abbreviation: enroll, enrollment/baseline; EOC2, end of cycle 2; PBMC, peripheral blood mononuclear cells.

C-C chemokine receptor type 7 (CCR7) is a marker for T cell chemotaxis and tumor tissue homing, with reduced expression as T cells become active. Baseline CCR7^+^CD3^+^ T cell levels were lower in patients with PDA vs. untreated controls and in tumors vs. periphery, and levels further decreased in both tumor and peripheral blood after treatment, consistent with T cell activation (Figure 4a). The major histocompatibility antigen class II (HLA-DR) is a ligand for the T cell receptor and correlates with T cell activation. Baseline HLA-DR^+^CD3^+^ T cells were increased in patients vs. controls, as well as in tumors vs. matched PBMCs, and levels increased slightly in both tumor and periphery after treatment (Figure 4a). PD-1 receptor on naïve CD3^+^ T cells is generally induced following TCR-mediated T cell activation, but constitutive expression by tumor-specific T cells is associated with other inhibitory receptors including TIM-3 and LAG-3 and contributes to T cell exhaustion [31,32]. We observed generally higher levels of PD-1, LAG-3 and TIM-3 on CD3^+^ T cells in tumors vs. periphery at baseline, and levels decreased or remained stable post-treatment (Figure 4b). Granzyme B is a critical mediator of cytotoxic T cell function, and CD25 and FoxP3 are markers of regulatory immune-suppressive CD3^+^ T cells. Granzyme B^+^ CD3^+^ T cells were significantly higher in PDA patients vs. untreated controls, and peripheral Granzyme B^+^ T cells further increased, whereas CD25^+^ and FoxP3^+^ T cells decreased post-treatment (Figure 4c).

There were no associations between baseline tumor CD8^+^ T cells, CD4^+^ T cells or any activated CD3^+^ T cell levels with OS or best response.

## 4. Discussion

Pancreatic ductal adenocarcinoma has a densely fibrotic stroma with high levels of hyaluronic acid accumulation [33]. It is thought that stromal extracellular matrix impedes perfusion by compressing the vasculature; thus, targeting stroma may improve drug delivery and allow effector immune infiltration. Several stroma-targeting strategies have been tested in PDA, some of which were biomarker-selected, such as PEGPH20 for HA-high PDA or peptide receptor radionuclide therapy (PRRT) and antibody drug conjugates (ADCs) for fibroblast-activating protein (FAP)-positive tumors [34]. Combinations of immunotherapy and stroma-targeting agents are of high interest due to observed preclinical synergism with improved cellular immune infiltration [21,22,35,36].

In this phase II study, we aimed to determine the safety and efficacy of PEGPH20 with pembrolizumab and assess whether stroma targeting by enzymatic degradation of HA enables immune cell infiltration and activation to increase efficacy from PD-1 checkpoint inhibition in HA-high refractory PDA. Due to the sponsors’ decision, this study closed to accrual prematurely after only eight of the planned 31 patients were enrolled. The combination of weekly PEGPH20 and pembrolizumab every 3 weeks was well-tolerated, and no patient stopped treatment for toxicity. While no patient responded, two patients with lung and liver metastases had SD (29%) lasting 2 and 9 months, respectively. The median PFS of 1.5 months was low, similar to other studies for refractory PDA [37,38], but the median OS of 7 months in patients pretreated with a median of two prior lines of therapy, most with liver (n = 5) and peritoneal (n = 5) metastases, was encouraging. Among four patients without rapid clinical deterioration who were treated post-study with chemotherapy, OS ranged from 10 to 27.6+ months, suggesting immune modulatory effects from combined stroma targeting plus immunotherapy or enhanced efficacy of subsequent chemotherapy after stromal depletion.

With the caveat of few on-study blood and tumor samples, we observed increased numbers of peripheral memory CD8^+^ and CD4^+^ T cells, as well as increased markers of T cell activation and decreased T cell exhaustion during treatment. One set of paired tumor biopsies noted TCR clonality expansion and increased CD8^+^ T cell levels post-treatment.

Few patients with PDA respond to immune checkpoint inhibitors, and MSI-high, TMB-high, or homologous recombination deficiency (HRD)-high tumors derive most benefit [12,13,39]. Hopkins et al. noted a correlation between lower baseline peripheral TCR clonality with long-term OS for PDA patients treated with anti-CTLA4 ICI and higher clonality post-treatment associated with long-term OS for PDA patients treated with anti-PD1 ICI [28]. In this study we observed higher baseline tumor TCR clonality and higher frequency of productive clones to be associated with longer OS. Premature study closure precluded any associations with post-treatment tumor TCR clonality. In addition, we did not find any correlations between peripheral TCR clonality and outcomes. These findings (Figure 3d,e) are concordant with other studies finding higher TCR repertoire clonality to correlate with improved patient outcomes in pancreatic cancer [40,41].

Recent data suggest that the likelihood of benefits from immunotherapy may be better characterized by the multidimensional topography of immune-infiltrating T cells in relation to tumor epithelial cells. Immunoscore-immune-checkpoint (IC) for example, measures densities and proximities between PD-L1^+^ cells and CD8^+^ T cells and has predicted benefits from ICI in MSS colorectal cancer [42] and non-small cell lung cancers [43]. Such robust profiling of the tumor immune contexture may provide insight into which immunologically cold tumors may benefit from treatment with ICI [44].

Ko et al. recently described the results of atezolizumab plus PEGPH20 compared to chemotherapy [Gem/nabP or folinic acid, 5-FU, oxaliplatin (FOLFOX)] in the MORPHEUS basket study for second-line treatment of patients with metastatic PDA, unselected for HA-high status [45]. ORR with atezolizumab plus PEGPH20 vs. chemotherapy was 6.1% vs. 2.4% and median PFS and OS were 1.5 months vs. 2.3 months and 7.1 vs. 6.8 months, respectively. Among patients with HA-high status, atezolizumab plus PEGPH20 conferred median OS of 7.9 months vs. 6.3 months with chemotherapy. We observed similar OS in a more heavily pretreated population in this study.

## 5. Conclusions

In summary, PEGPH20 plus pembrolizumab is safe but has limited efficacy in refractory HA-high pancreatic ductal adenocarcinomas. While no responses occurred, some patients treated post-study with chemotherapy had longer than expected survival, possibly related to host and tumor microenvironment immune modulatory effects. Biomarker studies demonstrated that cytotoxic and helper T cells, including activated memory T cells, are present in tumors and in the peripheral blood of advanced pancreatic cancers and may expand after treatment, as well as the fact that TCR clonality may correlate with survival. Small patient numbers, with rapid clinical deterioration for some patients, and premature study closure precluded robust correlative analysis. Nevertheless, targeting tumor stroma in combination with immunotherapy remains of significant interest in pancreatic cancer, and signals of preliminary activity exist, including in patients with *KRAS*-mutated tumors and with liver metastases. Biomarker discovery, both tumor- and host-related, to predict benefits is of critical need for precision immunotherapy in PDA.

## Figures and Tables

**Table 1:** Patients’ characteristics.

Characteristic	Number	%
Age (median, years)		68
Range		(60–73)
<65	1	12.5
≥65	7	87.5
Gender		
Female	1	12.5
Male	7	87.5
Race		
Asian	1	12.5
Black African American	0	0
White	7	87.5
ECOG performance status		
0–1	8	100
Sites of metastatic disease		
Liver	5	62.5
Lung	1	12.5
Lymph nodes	3	37.5
Peritoneum	5	62.5
Number of prior therapies		
Median (range)	2 (1–4)	
Prior therapies		
Gemcitabine/nab-Paclitaxel	6	75
FOLFIRINOX	4	50
FOLFOX	2	25
5-FU/leucovorin	1	12.5
Gemcitabine	1	12.5
RX-3117/nab-Paclitaxel	1	12.5

Abbreviations: ECOG, Eastern Cooperative Oncology Group.

**Table 2:** Treatment-emergent adverse events occurring in >1 patient and treatment-related adverse events in all patients (NCI-CTCAE v4.0).

Adverse Event	Grade 1 n (%)	Grade 2 n (%)	Grade 3 n (%)	Grade 4 n (%)	All Grades n (%)
**TRAE**					
dyspnea	0	1 (13)	0	0	1 (13)
edema limbs	2 (25)	0	0	0	2 (25)
fatigue	0	0	1 (13)	0	1 (13)
hypothyroidism	0	1 (13)	0	0	1 (13)
muscle cramps	1 (13)	1 (13)	0	0	2 (25)
myalgia	1 (13)	3 (38)	0	0	4 (50)
**TEAE**					
abdominal pain	0	0	2 (25)	0	2 (25)
diarrhea	2 (25)	0	0	0	2 (25)
edema limbs	2 (25)	0	0	0	2 (25)
muscle cramps	1 (12.5)	1 (12.5)	0	0	2 (25)
myalgia	1 (12.5)	3 (37.5)	0	0	4 (50)
vomiting	1 (12.5)	1 (12.5)	0	0	2 (25)

Abbreviations: TEAE, treatment-emergent adverse event; TRAE, treatment-related adverse event.

**Table 3:** Summary of molecular profile, prior and subsequent therapies, and outcomes for all patients.

Pt	Molecular Profile	PD-L1 MPS	TMB (m/Mb)	Prior Therapies	Best Response	PFS (mo)	Subsequent Therapies	OS (mo)
1	Not done	3		FFOX Gem/nabP Gem FFOX	NE	1.3	none	1.3
2	MSS, *KRAS* WT, *ATM* L1238fs*6, *RET*-*PCM1* fusion, *RNF43* R132 *	1	6	RX3117/nabP FOLFOX	PD	2.0	nal-iri/5FU	27.6+
3	Not done	1		Gem/nabP 5FU/LV	PD	1.0	none	2.1
4	MSS, *KRAS* G12R, *FGFR1* amp, *ZNF703* amp, *NSD3* amp, *SMAD4* 1309-1_1309GG > TTT, *TP53* V157D, *RB1* 1369fs*8, *PRKAR1A* R96 *	1	1	FFOX Gem/nabP	PD	1.2	nal-iri/5FU	11.9
5	Not done	1		FOLFOX FFOX	SD	10.2	Gem	10.2
6	Not done	0		Gem/nabP	SD	4.5	FOLFOX	11.2
7	MSS, *KRAS* G12D, *BRCA2* T207A, *TP53* R273L	NE	<10 *	Gem/nabP	PD	1.1	none	1.6
8	MSS, *KRAS* mut, *TP53* mut, *CDKN2A* mut, *CDH1* mut ^&^	2	<10 *	FFOX Gem/nabP	PD	1.7	none	4.5

Abbreviations: amp, amplification; cis, cisplatin; FFOX, FOLFIRINOX; Gem, gemcitabine; mo, months; MSS, microsatellite stable; MPS, modified proportion score includes tumor cells and mononuclear inflammatory cells; m/Mb, mutation per megabase; mut, mutation; nabP, nab-paclitaxel; nal-Iri, nano-liposomal irinotecan; NE, not evaluable; PD, progressive disease; Pt, patient; SD, stable disease; TMB, tumor mutational burden; WT, wild-type; ^&^: no details were available on specific genetic mutations. *: no discrete value was available for TMB for two patients, and low TMB < 10 m/mb was indicated in medical record.

## Data Availability

Trial data were collected, managed, stored, shared, and archived according to the Cancer Research and Biostatistics standard operating procedures to ensure the enduring quality, integrity, and use of the data. Formal requests for data sharing are considered with due regard given to funder and sponsor guidelines. Data will be available after this study’s publication. Requests are via a standard pro forma describing the nature of the proposed research and the extent of data requirements. Data recipients are required to enter a formal data sharing agreement that describes the conditions for release and requirements for data transfer, storage, archiving, publication, and intellectual property.

## Supplementary Materials

The following supporting information can be downloaded at: https://www.mdpi.com/article/10.3390/cancers18030507/s1, Supplementary Figure S1: Immune Phenotyping for (a) Cytotoxic CD8+T cells as a percentage of CD3+T cells from PBMCs and Tumors, (b) Memory CD45RO+/CD45RA- CD8+T cells in PBMCs and Tumors, and (c) Memory CD45RO+/CD45RA- CD4+T cells in PBMCs and Tumors.
