# Supplementary material for: Phase II Study of Pegvorhyaluronidase Alfa (PEGPH20) and Pembrolizumab for Patients with Hyaluronan-High, Pretreated Metastatic Pancreatic Ductal Adenocarcinoma: PCRT16-001
Source: Cancers (Basel). 2026 Feb 3;18(3):507. doi: 10.3390/cancers18030507 (PMC12896646; doi:10.3390/cancers18030507)
Supplement: Supplementary file 1 [file cancers-18-00507-s001.zip › cancers-4106319-supplementary.pdf]

Supplementary Materials

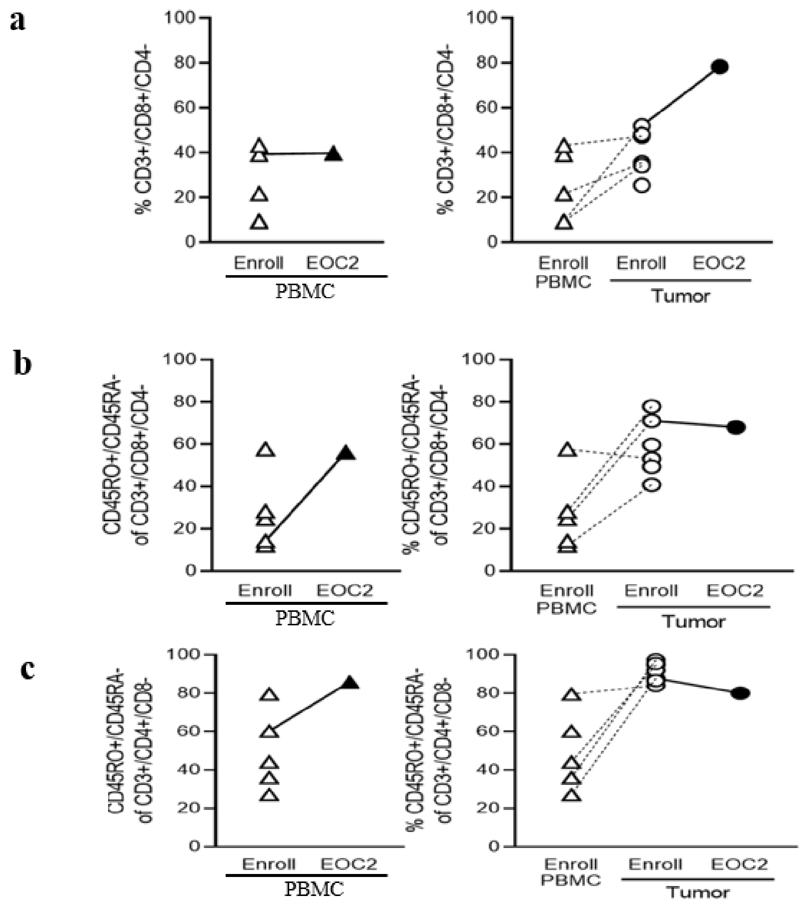

Supplementary Figure S1: Immune Phenotyping for (**a**) Cytotoxic $CD8^+$T cells as a percentage of $CD3^+$T cells from PBMCs and Tumors, (**b**) Memory $CD45RO^+/CD45RA^-$ $CD8^+$T cells in PBMCs and Tumors, and (**c**) Memory $CD45RO^+/CD45RA^-$ $CD4^+$T cells in PBMCs and Tumors

Abbreviations: Enroll, at enrollment/baseline; EOC2, end of cycle 2; PBMCs, peripheral blood mononuclear cells
